# STK3-ALK, a Novel ALK Rearrangement in Non-Small Cell Lung Cancer With Sensitivity to Tyrosine Kinase Inhibitors: A Case Report

**DOI:** 10.3389/fonc.2021.700341

**Published:** 2021-08-19

**Authors:** Chunlai Feng, Rong Zhou, Feng Liu, Tingting Wang, Sisi Liu, Yang Shao

**Affiliations:** ^1^Department of Respiratory Medicine, The Third Affiliated Hospital of Soochow University, Changzhou, China; ^2^Department of Research and Development, Nanjing Geneseeq Technology Inc., Nanjing, China

**Keywords:** *ALK* rearrangement, NSCLC, *STK3-ALK*, brain metastases, TKI, case report

## Abstract

Anaplastic lymphoma kinase (ALK) rearrangement occurs in 5% to 8% of patients with non-small cell lung cancer (NSCLC). More than 90 different ALK fusion partners have been discovered in NSCLC patients, and ALK tyrosine kinase inhibitors (TKIs) such as crizotinib and alectinib have achieved tumor responses in patients with advanced ALK-positive NSCLC. Here, we report the case of a patient with an advanced NSCLC carrying a novel serine/threonine kinase 3 (STK3)-ALK rearrangement, which was identified by targeted next-generation sequencing (NGS) and was confirmed by RNA sequencing. Anti-ALK immunohistochemistry (IHC) staining also revealed the high expression of ALK. The patient benefitted from alectinib treatment after experiencing crizotinib resistance and achieved an overall response to TKI of over 14 months. At the timepoint of submission of this manuscript, this patient is still receiving alectinib treatment with a good tolerance. This study provides meaningful insights into the potential treatment option for NSCLC patients with brain metastases harboring *STK3-ALK* fusions and highlights the advantages of NGS in rapidly identifying novel molecular targets.

## Introduction

Anaplastic lymphoma kinase (*ALK*) rearrangement has been identified in up to 8% of non-small cell lung cancer (NSCLC) cases (1). As of the end of January 2020, over 90 distinct ALK fusion partners have been discovered in NSCLC patients (2). As *ALK*-rearranged NSCLC patients respond to ALK tyrosine kinase inhibitors (TKIs), such as crizotinib, alectinib, and lorlatinib (3, 4), the identification of druggable ALK fusions is crucial for NSCLC treatment. Compared to traditional methods such as PCR and immunohistochemistry, next-generation sequencing (NGS) provides more comprehensive genomic information of cancers and increases the pace of identifying novel ALK fusions (2). Here, we report a patient with lung adenocarcinoma, harboring a novel serine/threonine kinase 3 (STK3)–ALK fusion, who was treated with crizotinib and alectinib and achieved an overall response of over 14 months. Our findings provide meaningful insights into the understanding of genetic profiling associated with tumors and underscore the importance of genetic testing in revealing novel druggable genetic mutations.

## Case Report

**Figure 1:**
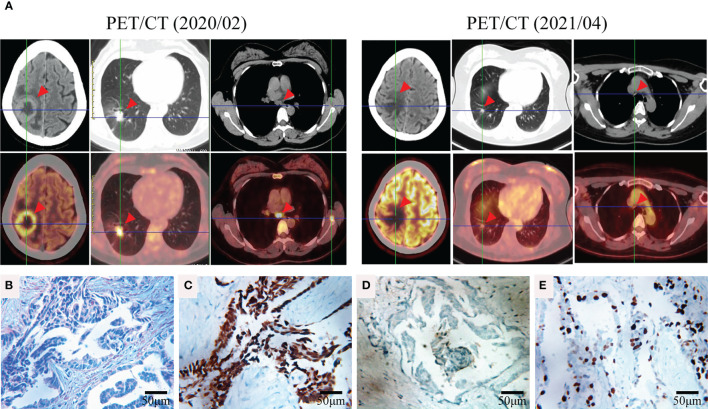
Representative clinical images obtained during the course of treatment. **(A)** FDG-PET/CT of the brain, lung, and mediastinum before (2020/02) and after (2021/04) TKI therapy. **(B)** Hematoxylin and eosin (HE) staining of the tumor biopsy revealed a few atypical glands. Immunohistochemical staining of thyroid transcription factor (TTF1) **(C)**, p40 **(D)**, and Ki-67 **(E)** of the tumor biopsy (400×).

A 42-year-old female patient was admitted to a hospital in February 2020 following 2 months of intermittent, nonproductive cough. The patient was a hairdresser who had a long history of passive smoking exposure. Chest computed tomography (CT) and positron emission tomography (PET)-CT scanning revealed multiple tumor lesions in the inferior lobe of the right lung and the occipital lobe of the brain, which was clinically suggestive of lung cancer and brain metastases (BMs) (**Figure 1A**). Histological analysis of the tumor biopsy showed a few atypical glands (**Figure 1B**), and immunohistochemistry staining (IHC) of the tumor biopsy revealed remarkable positivity for thyroid transcription factor (TTF1) and Ki-67 (50%) (**Figures 1C, E**) and negativity for p40 (**Figure 1D**). Collectively, the results suggested the presence of stage IVB lung adenocarcinoma (T1cN3M1c).

**Figure 2:**
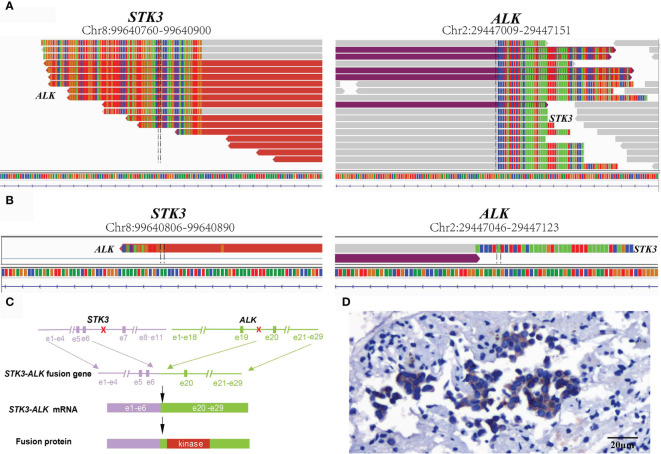
Sequence analysis of the *STK3-ALK* fusion. **(A)** DNA and **(B)** RNA-level fusion reads of *STK3-ALK* are shown from the Integrative Genomics Viewer. **(C)** A schematic map showing the structure of the STK3-ALK fusion locus. Exons 1–6 of STK3 (purple) were fused to exons 20–29 of ALK (green) through intron6 of STK3 and intron19 of ALK. **(D)** Immunohistochemical staining revealed the diffuse expression of ALK in tumor cells (20×).

**Figure 3:**
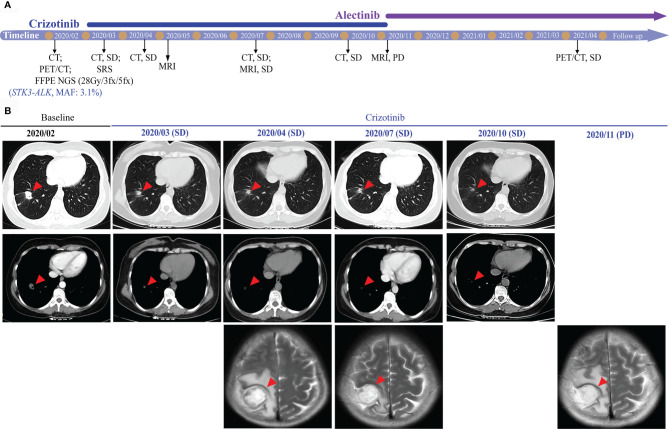
Radiological features before and after crizotinib therapy. **(A)** Timeline of therapies for advanced NSCLC. **(B)** The chest CT and brain MRI scans reveal the control of disease after crizotinib treatment. Tumors are indicated by red arrows. CT, computed tomography; MRI, magnetic resonance imaging; SRS, stereotactic radiosurgery; MAF, mutant allele frequency; SD, stable disease; PD, progressive disease.

For a comprehensive molecular profiling of targetable genetic alterations, targeted NGS of 139 lung cancer–related genes (Geneseeq LungTrak) from the tumor biopsy was performed (5). The turnaround time for NGS for lung cancer samples is less than 7 days. Interestingly, a novel *STK3-ALK* rearrangement was identified with a mutant allele frequency of 3.1% (**Figure 2A**). No other targetable mutations were observed. RNA sequencing (RNA-seq) confirmed the novel fusion (**Figure 2B**), and anti-ALK immunohistochemistry revealed a high level of ALK protein expression (**Figure 2D**). Therefore, crizotinib (250 mg, bid) plus stereotactic radiosurgery (SRS, 28Gy/3fx/5fx) to the brain was recommended as the first-line treatment beginning in March 2020 (**Figure 3A**). One month after treatment, a chest CT scan demonstrated a slightly reduced primary tumor mass, which indicated a stable disease (SD) according to the Response Evaluation Criteria in Solid Tumors (RECIST 1.1) guideline. In July 2020, a CT re-examination revealed a stable primary tumor, and brain magnetic resonance imaging (MRI) showed slightly decreased tumor size in brain. Unfortunately, after 8 months of crizotinib therapy, an MRI scan revealed progressive disease (PD), manifesting as central nervous system (CNS) progression (**Figure 3B**). Subsequently, after developing crizotinib resistance, the patient was administered alectinib (600 mg, bid) in November 2020. The PET-CT scan in April 2021 revealed decreased tumor lesions in her right lung and brain which indicated an SD (**Figure 1A**). The patient is currently receiving alectinib treatment with good tolerance.

## Discussion

ALK rearrangements are among the most common driver mutations in lung cancer and can be targeted by ALK TKIs (6). Herein, we reported a novel *STK3-ALK* fusion that was identified by targeted-NGS and benefitted from TKI treatment. STK3, also named MST2, is a key component of the Hippo signaling network and comprises a kinase domain (amino acids 1–297), an inhibitory domain (amino acids 327–392), and a C-terminal SARAH domain (amino acids 436–491) (7). *STK3* is a growth suppressor encoding a serine/threonine protein kinase activated by proapoptotic molecules (8). In this case, the STK3-ALK fusion was produced by a translocation event that fused the ALK kinase domain on chromosome 2, with the partial kinase domain of the STK3 gene on chromosome 8, linking intron 6 of STK3 to intron 20 of ALK (**Figure 2C**). The *STK3-ALK* fusion may lead to the stabilization and activation of the ALK protein and eventually drive oncogenesis. Further studies should be conducted to confirm the biological function of the STK3-ALK fusion in tumorigenesis. The ALK gene rearrangements present in lung adenocarcinoma typically involved a 5’ fusion of the echinoderm microtubule-associated protein-like 4 (EML4) gene to the ALK kinase domain. NSCLC patients who express a transforming fusion kinase can benefit from treatment with ALK-TKIs (9). Herein, a STK3-ALK fusion harboring an ALK kinase domain was identified as a promising candidate for a therapeutic target.

Nearly 20% of NSCLC patients are diagnosed with BMs (10, 11). Surgical resection (SR), SRS, and whole-brain radiation therapy (WBRT) are the traditional primary treatments for NSCLC patients with BMs (12). Among such approaches, SRS is the most common with a longer cognitive-free deterioration survival and shorter time to intracranial disease progression compared to WBRT (13). BMs are also a challenge for ALK-positive cancer patients who have a cumulative risk of developing CNS disease of 70% within 5 years of diagnosis (14).

Crizotinib is the first ALK-TKI approved by the Food and Drug Administration in 2011 for ALK-positive NSCLC patients (15). It has been reported that *ALK-*rearranged NSCLC patients with BMs have prolonged survival following treatment with brain radiotherapy and crizotinib (16). Compared to patients receiving crizotinib, patients receiving alectinib (second-generation TKI) or lorlatinib (third-generation TKI) showed better CNS efficacy and had significantly longer progression-free survival (PFS) and a higher frequency of intracranial response in advanced ALK-positive NSCLC (4, 17, 18). Despite the level of CNS activity, lorlatinib had a higher incidence of grade 3 or 4 adverse events (19). According to the National Comprehensive Cancer Network (NCCN) guidelines, patients with BMs who progressed on crizotinib could benefit from alectinib plus local therapy (12). Therefore, the patient was treated with alectinib after developing crizotinib resistance and showed a good tolerance for over 5 months. In fact, the patient still receives alectinib treatment and is regularly followed up.

Hairdressers who are exposed to a wide range of chemicals have a higher risk of developing cancers than the general population (20). Follow-up studies on the carcinogenic risks of hairdressers and users of hair dyes have revealed an increased risk of skin cancers of the scalp and neck, as well as lung cancer (21). In one study, the pooled relative risk (RR) of hairdressers developing lung cancer was 1.27 (95% CI 1.15–1.41) (22). Herein, we reported a hairdresser who was diagnosed with lung adenocarcinoma. The relationship between hair dye and the risk of lung cancer should be evaluated in future studies.

## Conclusions

In summary, we detected a novel STK3-ALK fusion in an NSCLC patient with BM who benefitted from TKI therapy and SRS. This study provides meaningful insights into the potential treatment option for NSCLC patients whose tumors harbor STK3-ALK fusions and highlights the advantage of NGS in rapidly revealing novel molecular targets of diseases.

## Data Availability Statement

The data presented in the study are deposited in the China National Genebank (CNGB, https://db.cngb.org/cnsa/), the accession number is CNP0002024.

## Ethics Statement

The study protocol was approved by ethical committee of Department of Respiratory Medicine, The Third Affiliated Hospital of Soochow University. The patients/participants provided their written informed consent to participate in this study.

## Author Contributions

CF: Conceptualization and data curation. RZ and FL: Writing-original draft preparation. TW: Edited the figures. SL and YS: Writing-review and editing. All authors contributed to the article and approved the submitted version.

## Funding

This work was supported by the Foundation of Municipal Health and Family Planning Commission in Changzhou [grant number ZD201603].

## Conflict of Interest

TW, SL, and YS are employees of Nanjing Geneseeq Technology Inc., China.

The remaining authors declare that the research was conducted in the absence of any commercial or financial relationships that could be construed as a potential conflict of interest.

## Publisher’s Note

All claims expressed in this article are solely those of the authors and do not necessarily represent those of their affiliated organizations, or those of the publisher, the editors and the reviewers. Any product that may be evaluated in this article, or claim that may be made by its manufacturer, is not guaranteed or endorsed by the publisher.
